# Lipid Biomarkers in Liquid Biopsies: Novel Opportunities for Cancer Diagnosis

**DOI:** 10.3390/pharmaceutics15020437

**Published:** 2023-01-28

**Authors:** Krizia Sagini, Lorena Urbanelli, Sandra Buratta, Carla Emiliani, Alicia Llorente

**Affiliations:** 1 Department of Molecular Cell Biology, Institute for Cancer Research, Oslo University Hospital, The Norwegian Radium Hospital, 0379 Oslo, Norway; 2 Centre for Cancer Cell Reprogramming, Faculty of Medicine, University of Oslo, Montebello, 0379 Oslo, Norway; 3 Department of Chemistry, Biology and Biotechnology, University of Perugia, 06123 Perugia, Italy; 4 CEMIN (Center of Excellence for Innovative Nanostructured Material), University of Perugia, 06123 Perugia, Italy; 5 Department for Mechanical, Electronics and Chemical Engineering, Oslo Metropolitan University, 0167 Oslo, Norway

**Keywords:** biofluids, biomarkers, cancer, extracellular vesicles, lipid metabolism, lipidomics, liquid biopsy, mass spectrometry

## Abstract

Altered cellular metabolism is a well-established hallmark of cancer. Although most studies have focused on the metabolism of glucose and glutamine, the upregulation of lipid metabolism is also frequent in cells undergoing oncogenic transformation. In fact, cancer cells need to meet the enhanced demand of plasma membrane synthesis and energy production to support their proliferation. Moreover, lipids are precursors of signaling molecules, termed lipid mediators, which play a role in shaping the tumor microenvironment. Recent methodological advances in lipid analysis have prompted studies aimed at investigating the whole lipid content of a sample (lipidome) to unravel the complexity of lipid changes in cancer patient biofluids. This review focuses on the application of mass spectrometry-based lipidomics for the discovery of cancer biomarkers. Here, we have summarized the main lipid alteration in cancer patients’ biofluids and uncovered their potential use for the early detection of the disease and treatment selection. We also discuss the advantages of using biofluid-derived extracellular vesicles as a platform for lipid biomarker discovery. These vesicles have a molecular signature that is a fingerprint of their originating cells. Hence, the analysis of their molecular cargo has emerged as a promising strategy for the identification of sensitive and specific biomarkers compared to the analysis of the unprocessed biofluid.

## 1. Introduction

Altered cellular metabolism is a well-established hallmark of cancer [1]. The first observation of cancer metabolic alterations was made in the 1920s by Otto Warburg. He noticed that cancer cells use large amounts of glucose to generate lactate, even in the presence of oxygen, a phenomenon termed aerobic glycolysis or the Warburg effect [2]. Since then, our understanding of tumor-associated metabolic alterations, underlying molecular mechanisms and functional consequences in tumorigenesis has expanded. A variety of well-known oncogenes, such as c-myc, AKT, mTOR, hypoxia-inducible factors and RAS, have been shown to contribute to the metabolic adaptations of cancer cells [3]. Multiple studies have uncovered how glycolysis and the tricarboxylic acid cycle generate metabolic intermediates that sustain the de novo synthesis of nucleotides, lipids and amino acids, supporting cancer cell proliferation [4]. Moreover, some metabolites, known as oncometabolites, accumulate in cancer cells in response to an altered expression of metabolic enzymes and have been directly linked to tumor growth. A canonical example is the cancer-associated mutations in isocitrate dehydrogenase 1 and 2, which result in the aberrant production of the oncometabolite 2-hydroxyglutarate (2HG). The cellular accumulation of 2HG inhibits enzymes controlling histone and DNA demethylation, thus altering the chromatin landscape and gene expression [5]. The majority of studies so far have focused on the metabolism of glucose and glutamine, but it is becoming clear that lipid metabolism is also frequently altered in cells undergoing oncogenic transformation [6]. Cancer cells often up-regulate de novo lipogenesis, fatty acid (FA) uptake, FA oxidation (FAO) and lipid accumulation to support cellular proliferation and the consequent higher demand of plasma membrane synthesis and energy production [7]. Improved lipid analysis techniques based on liquid/gas chromatography coupled with mass spectrometry (MS) have enabled quantitative profiling of lipids as individual molecular species from minute amounts of samples, allowing the in-depth characterization of the lipidome [8]. These technologies allow for obtaining cancer-specific lipid profiles, which reflect, at a phenotypic level, molecular alterations and environmental factors (such as exposure to high-fat diets). These profiles may help in unraveling novel therapeutic targets and biomarkers for the early detection of cancer, possibly leading to improvements in the clinical strategy to treat the disease.

In this review, we present crucial aspects of lipid metabolism associated with cancer. We also describe recent findings highlighting lipidomic alterations in cancer patients’ biofluids and biofluid-derived extracellular vesicles (EVs), thus uncovering the application of lipidomics in liquid biopsy.

## 2. Lipidic Metabolism in Cancer

Lipids represent a complex group of biomolecules that vary in structure and perform three main functions: energy storage, membrane formation and signaling. The majority of lipids derivates from FAs, molecules consisting of hydrocarbon chains varying in length and saturation.

Triglycerides (TAGs) are formed by a glycerol moiety esterified with three FAs and, in cells, are localized within lipid droplets. They represent the main storage form of lipids for energetic purposes, as TAG-derived FAs in mitochondria can be catabolized by FAO for ATP production. Overexpression of FAO enzymes, such as the rate-limiting enzyme carnitine palmitoyltransferase 1 and acyl-CoA synthetase long-chain 3 (ACSL3), has been found in numerous malignancies and has been correlated to tumor growth, especially in adverse environmental conditions, such as glucose deprivation [9]. In some cancers, FAO is activated by specific oncogenes, such as c-Myc or mutant K-Ras [7].

**Figure 1:**
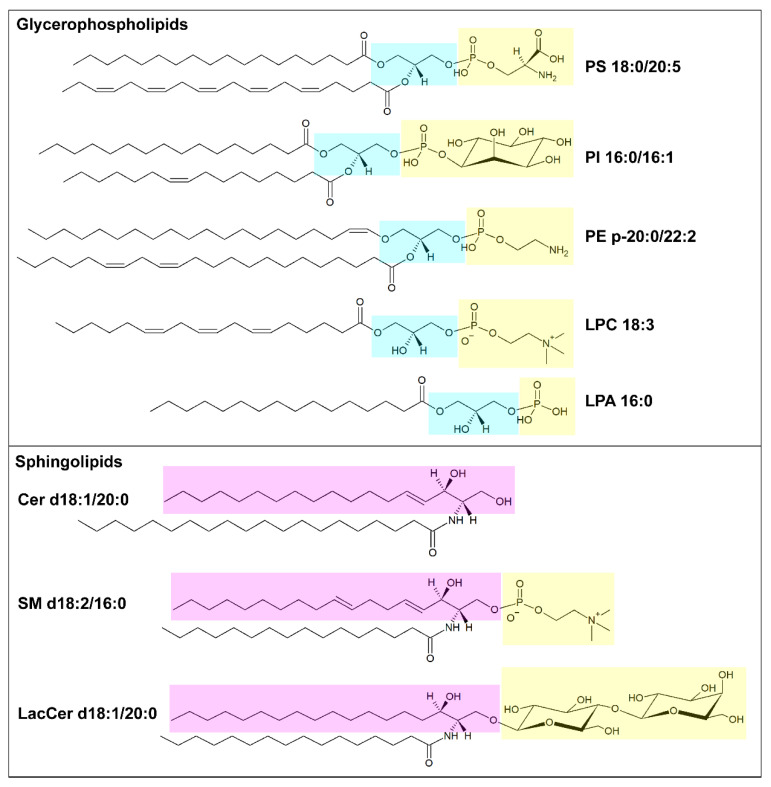
Schematic illustration of membrane lipids. In the upper box, example of glycerophospholipids: phosphatidylserine (PS) 18:0/20:5, phosphatidylinositol (PI) 16:0/16:1, alkenyl ether (plasmalogen) phosphatidylethanolamine (PE) p20:0/22:2, lysophosphatidylcholine (LPC) 18:3 and lysophosphatidic acid (LPA) 16:0. The glycerol moiety is marked in light blue, and the polar head is marked in yellow. In the lower box, examples of sphingolipids: ceramide (Cer) d18:1/20:0, sphingomyelin (SM) d18:2/16:0 and lactosylceramide (LacCer) d18:1/20:0. The pink box highlights the long-chain base (LCB), sphingosine, and the yellow one highlights the polar head. Structures have been made using the Structure Drawing Tools available at Lipid Maps [15].

Lipids organized in lamellar bilayers form the architecture of biological membranes. The main membrane constituents in mammals are phospholipids and cholesterol (Table 1). Phospholipids can be further classified according to their structure into glycerophospholipids (consisting of glycerol bound to two fatty acyl chains and a polar head formed by a phosphate group linked to a polar group) and sphingolipids (consisting of ceramide and a polar head formed by a phosphate group linked to choline or ethanolamine) (Figure 1). Glycerophospholipids carry different polar groups, giving rise to various lipid classes: phosphatidic acid (PA), phosphatidylcholine (PC), phosphatidylserine (PS), phosphatidylethanolamine (PE), phosphatidylglycerol (PG) and phosphatidylinositol (PI). Although most glycerophospholipids contain ester-bound FAs, ether-linked fatty acyl chains are also common [10]. Sphingolipids also can have different head groups and are therefore classified into ceramide (Cer, consisting of a long-chain base, often sphingosine, linked to a FA via the amino group) and complex sphingolipids such as sphingomyelin (SM) and glycosphingolipids (glucosylceramides, GlcCer; galactosylceramides, GalCer; sulfatides, Sulf; lactosylceramides, LacCer; gangliosides, GM). The lipid bilayer of the plasma membrane has an asymmetric distribution of lipids. Its outer leaflet contains mostly PC and sphingolipids, and the inner leaflet contains PE, PS, and PI, whereas cholesterol is more evenly distributed between the two leaflets. Similar asymmetry is also often found in other organelle membranes. The fatty acyl chains in glycerophospholipids mostly contain 16 or 18 carbon atoms and zero or few double bonds in the cis configuration. Nevertheless, longer polyunsaturated FAs (PUFAs), such as arachidonic acid (AA, C20:4), eicosapentaenoic acid (EPA, C22:5) and docosahexanoic acid (DHA, C22:6), are frequently bound to the sn-2 position [9]. In sphingolipids, instead, a marked difference in acyl chain length, often with C16:0 as the shortest species and C22-C24 as the longest species, is common [11]. The saturation degree of membrane lipids regulates membrane fluidity and cell homeostasis. Consistently, the accumulation of lipids containing saturated FAs can lead to endoplasmic reticulum stress and apoptosis [12], while high amounts of PUFAs sensitize cells to lipid peroxidation and ferroptosis, a non-apoptotic iron-dependent form of cell death [13]. The relative abundance of saturated and unsaturated FAs in membrane phospholipids primarily depends on FA availability and can be regulated by FA remodeling. In particular, the Lands’ cycle consists of a series of deacylation at the sn-2 position of glycerophospholipids by phospholipase A2 followed by reacylation by lysophospholipid acyltransferases (LPLATs) [14]. As different LPLAT isoforms differ in their affinity for FAs and lysophospholipids, this process generates diverse phospholipid species.

The de novo synthesis of FAs is low in normal adult cells (except for lipogenic tissues, such as liver and adipose tissue). In contrast, increased lipogenesis is well documented in cancer cells, making them less dependent on the availability of nutrients and enabling the building of a cell membrane enriched in phospholipids containing oxidative damage-resistant saturated FAs [16]. The biosynthesis of FAs begins with the carboxylation of cytosolic acetyl-CoA by acetyl-CoA carboxylase (ACC) to produce malonyl-CoA, which becomes the substrate of FA synthase (FASN), forming palmitic acid (C16:0). Palmitate can be further elongated by FA elongases (ELOVL1-7) and desaturated by stearoyl-CoA desaturases (SCD) and FA desaturases (FADS1-3), to generate a cellular pool of non-essential FAs, such as palmitoleate (C16:1), stearate (C18:0) and oleate (C18:1). All three enzyme families involved in FA synthesis (ACC, FASN, SCD) are frequently up-regulated in several cancer types, including glioblastoma, breast, ovarian, lung, prostate and liver cancer; furthermore, their expression correlates with poorer prognosis and high cancer grade [7]. To be incorporated into membrane lipids, free FAs need to be activated to their corresponding acyl-CoA by acyl-CoA synthetases (ACS). Among ACS family members, characterized by different lengths of fatty acyl chains used as substrates, a relevant role in cancer cells has been assigned to long-chain ACS (ACSL), which activates FAs formed by 12 to 20 carbon atoms. ACSLs exhibit distinct substrate preferences, e.g., ACSL4 has a strong preference for PUFAs, and ACSL3 conjugates both PUFAs and monounsaturated FAs (MUFAs). Therefore, by regulating the activity of ACSLs, cancer cells can control the saturation degree of their membrane [17]. Various oncogenic signals, such as the PI3K/AKT/mTORC1 axis, BRAF and c-Myc, have been implicated in the upregulation of lipogenesis by activating sterol regulatory element-binding protein 1 (SREBP1), the major regulator of genes involved in FA synthesis [18,19,20]. Moreover, in some cases, cancer cells may increase the availability of free FAs by increasing TAG degradation. Hence, a higher level of monoacylglycerol lipase has been detected across various aggressive cancer types [21].

Mammals can only produce certain FAs, whereas others, such as linoleic acid (C18:2 n6) and alpha-linolenic acid (C18:3 n3), are essential and must be taken up from the diet. Cells can uptake FAs through multiple routes, including receptor-mediated endocytosis of low-density lipoproteins (LDLs) or free FA import via membrane FA transporters, such as the FA translocase CD36. In particular, elevated CD36 levels and the increased uptake of free FAs have been correlated with enhanced metastasis formation and aggressiveness in oral cancer [22]. Moreover, tumor cells can increase the availability of Fas by inducing, in neighboring adipocytes, lipolysis and mobilization of Fas via the overexpression on their surface of the FA-binding protein FABP4 [23]. Metastatic cancers, including ovarian, breast and colorectal cancer [7], are also able to promote systemic adipose tissue atrophy in a TNF-α- and IL-6-dependent manner, which may result in cancer-associated cachexia [24].

Apart from their structural and energetic functions, lipids are important for cell signaling and protein trafficking. For instance, the peculiar composition of lipid rafts (i.e., sphingolipid- and cholesterol-rich plasma membrane microdomains) can aid the clustering of receptors, such as tyrosine kinase receptors, and their downstream signaling [25,26]. Moreover, membrane lipids can act as precursors for signaling molecules. Phosphoinositides (PIPs) are phosphorylated derivates of PI that help in specifying organelle identity and intracellular transport by recruiting cytosolic proteins containing specific recognition domains [27]. Phospholipase C-mediated hydrolysis of PIPs generates two important second messengers, diacylglycerol (DAG) and inositol trisphosphate, which are implicated in multiple signal cascades [28]. PLAs release free FAs from the sn-1 and sn-2 position of phospholipids, creating lysophospholipids that can be converted into lysoPA (LPA) via cleavage of the polar group by lysophospholipase D. LPAs might bind to G protein-coupled receptors, triggering the activation of different signaling axes, such as AKT signaling, to promote cell migration and survival [29]. Interestingly, the lysophospholipase D autotaxin has recently been shown to activate a stroma-cancer signaling axis that promotes tumor progression in pancreatic cancer [30]. Moreover, phospholipid-derived free FAs may serve as precursors for lipid mediators themselves, such as in the case of AA, the substrate for the synthesis of proinflammatory eicosanoids via the cyclooxygenase pathway. One of these, prostaglandin E2 has been implicated in the establishment of a tumor-promoting microenvironment by inducing cancer cell proliferation, migration and angiogenesis [31].

In summary, lipids play an important role in tumor development, sustaining cell proliferation and metastasis formation. Dysregulated lipid metabolism is not only a key component in cancer metabolic adaptation but also creates an altered lipid profile that can distinguish tumors from normal tissues. For this reason, lipidomics may add an additional layer of information to proteomics and genomics, expanding our knowledge of lipid functions and opening the path to new opportunities for drug and biomarker development.

## 3. Liquid Biopsies in Cancer

In recent years, the profiling of biofluid for disease status, also known as liquid biopsy, has become a hot topic in cancer clinical research. In fact, while tissue biopsies are invasive, costly and hardly representative of the tumor heterogeneity, liquid biopsies are nontraumatic, easy to obtain, reflect the overall state of the tumor, and allow real-time monitoring. As cancer cells release many factors, including lipids, that may be reminiscent of cellular metabolic and genetic alterations, the possibility of profiling these molecules in biofluids, possibly in an early phase, is extremely attractive. Moreover, liquid biopsies also facilitate the discovery of biomarkers originating from other cell types present in the tumor microenvironment (stromal cells, immune cells, fibroblasts and endothelial cells).

So far, liquid biopsy has mostly focused on the analysis of the genome, transcriptome and proteome, for which established methodologies have been long available. For instance, the US Food and Drug Administration has already approved biomarker tests for various cancer types based on the evaluation of genomic mutations and copy number alterations in cell-free DNA (cfDNA) [32]. Nevertheless, while the clinical relevance of cfDNA for monitoring metastatic disease is well demonstrated, the role of these biomarkers in early-stage patients remains to be established. Furthermore, the analysis of cell-free RNAs (cfRNAs) revealed that miRNAs are surprisingly stable in plasma or serum [33]. This prompted studies aimed at identifying miRNA signatures in biofluids to diagnose different cancer types. Proteomics has been recognized as a fundamental method for novel biomarker discovery, and a small number of protein markers, such as prostate-specific antigen (PSA) [34], are routinely used in clinics. However, for most proteins, information about cancer specificity is largely missing [35]. The more accurate discrimination of tumor types, stage and molecular subtypes may be achieved through multiparameter strategies, which combine information from multiple analyses. These will allow a more comprehensive understanding of cancer-associated molecular aberrations and facilitate the further establishment of liquid biopsies in the clinic.

Given the metabolic alterations associated with tumor development, the level of circulating metabolites, including lipids, has the potential to become an important source of cancer biomarkers. Lipidomics provide information on the lipid metabolism stage at a given time point. Recent advances in MS methodology, allowing lipid content analysis with extensive coverage and a high degree of sensitivity, have prompted studies aimed at using lipidomics to unravel lipid changes in disease [36]. Nevertheless, the chemical complexity of lipids, which have a wide structural diversity and different polarity ranges, poses challenges in performing comprehensive lipid profiling. Different extraction and analytic methods need to be used in order to be able to detect both polar and neutral lipids. Moreover, while MS-based protein identification is facilitated by known protein cleavage patterns, no fragmentation rules have been published for lipids, making their identification more complicated. It is also important to mention that analyte identification is often software-assisted, and several available databases are used to compare the experimental data to theoretical patterns. Due to incomplete/noisy databases, caution should be used to avoid mismatches and false positives.

Altogether, nucleic acid, protein and lipid biomarkers have advantages and disadvantages, and it is possible to foresee that a combination of multiple and/or combined approaches would possibly guarantee a further step in liquid biopsies. It must be considered that the advantages/disadvantages of these molecules as biomarkers are also dependent on the development of a technological platform suitable for their detection in an easy and reproducible manner (Table 2).

In recent years, EVs have emerged as a new source of biomarkers in liquid biopsies. EVs are a heterogenous family of micro-/nano-sized vesicles released by all cell types and retrieved in body fluids (i.e., plasma, urine, cerebrospinal fluid). They have a molecular signature that is a fingerprint of their originating cells and mirrors their physio-pathological state [37]. Cancer-derived EVs can be isolated from biofluids such as plasma/serum and urine [38]. Therefore, the analysis of their molecular cargo might represent a starting point for the discovery of sensitive and specific biomarkers.

In the following sections, we will review studies addressing the lipidic analysis of biofluids and biofluid-derived EVs, uncovering their potential application in cancer liquid biopsies.

## 4. The Lipidome of Biofluids as a Source of Potential Cancer Biomarkers

To make the sample collection for biomarker analyses simple, reproducible, and minimally invasive—all features necessary to develop a method for efficient population screening and monitoring—many studies have focused on the lipidomic analysis of easily accessible peripheral biofluids, such as blood derivatives (plasma and serum), and urine. It has been shown that plasma contains many thousands of distinct lipid species and that sterols are the most abundant (nmol/mL), followed by triglycerides, glycerophospholipids, free FAs, sphingolipids, and prenols [39]. Studies directed by the LIPID MAPS consortium have determined the consensus concentrations of over 500 lipid species in standard reference material (SRM 500), i.e., pooled human plasma obtained from healthy individuals with gender and ethnic balance, which may be used for comparative studies [39]. Rockwell et al. have reported the reproducible quantitation of over 600 lipid species belonging to 20 lipid classes from 0.5 mL of urine [40]. Using an MS/MS shotgun approach, they were able to identify free FAs and glycerophospholipids as the most abundant lipid classes in urine. Few pioneering studies have investigated the lipid profile of saliva and tears. The majority of salivary lipids comprise cholesterol, cholesterol esters, mono-, di- and triglycerides and free FAs [41]. It has also been shown that, among the more than 600 lipid species reported in tear fluid, cholesterol esters, waxes and phospholipids were the most abundant [42].

In this section, we are going to summarize the most relevant findings regarding the application of untargeted and targeted lipidomic strategies for the analysis of biofluids in different cancer types (Table 3). We will also underline the potential role of lipids as biomarkers, as well as focus on the current limits of these studies and possible strategies to overcome them.

### 4.1. Breast Cancer

Breast cancer (BC) is the most diagnosed life-threatening cancer in women [43], and early, non-invasive diagnosis is needed to early differentiate benign and malignant lesions, avoiding expensive and invasive screening. Over the last decades, a few studies investigated the whole plasma, serum and urine lipid content in BC patients. Qiu et al. applied an MS-based quantitative method to analyze plasma samples from 55 BC patients and 25 healthy controls [44]. They identified 39 differentiating species and reported significantly lower levels of lysoPCs (LPCs) and higher levels of SMs in the plasma samples obtained from BC patients, as compared with healthy controls. Moreover, three species (LPC 16:0, alkyl PC O-42:5, PC 34:2) successfully differentiated BC patients from healthy controls. Chen et al. investigated the lipid profile of a total of 194 plasma samples from 84 patients with early-stage BC (stage 0–II) and 110 patients with benign breast disease [45]. Authors reported that a combination of 15 lipid species could be used for the diagnosis of BC at an early stage. In another study carried out on a small number of plasma samples (6 benign breast tumors, 5 BC and 9 healthy controls), the authors found that two PI species (PI 32:1, PI 38:4) could differentiate between benign and malignant breast tumors [46]. The comparison of these three studies on plasma samples shows that the identification of candidate biomarkers does not converge on common lipid species. This happens when a very limited number of samples is analyzed [46], but also in studies using a more adequate number of individuals, although in this case, abnormal lipid levels of different species were identified at least in the same phospholipid subclasses, i.e., LPC and PC [44,45].

A few studies were also carried out using serum instead of plasma as the biological matrix. In 2017, More et al. reported the comprehensive lipidome of 28 malignant and benign breast tumor patients and healthy controls [47]. Results identified a few differentially expressed phospholipids (PE 30:1, PE 42:2, PC 36:0, and lysoPE (LPE) 14:0) discriminating BC from benign and healthy controls. A pilot study investigated the serum lipidomic signature of patients with confirmed invasive ductal carcinoma at three different stages (3 in stage I, 17 in stage II and 11 in stage III) [48]. The authors reported that BC evolution from stage I to III may be predicted by a few lipid species, with DAG 38:3 and DAG 34:5 showing the most relevant scores. Studies on serum were conducted on a more limited number of samples and focused on PEs [47] or DAGs [48] rather than the choline-containing phospholipids identified in two out of three studies on plasma [44,45]. In summary, these studies on BC blood derivatives do not converge in identifying common altered species. In addition, studies with a low number of samples are even less consistent. For this reason, analyses must be carried out with an appropriate number of samples, as a minimum inclusion of 30 subjects is highly recommended to provide minimal statistical power [49]. In addition, reducing as much as possible the variables associated with the collection, storage, and processing of blood samples, as well as the characteristics of study participants (i.e., age, body mass index, diet, medication, gender), is also recommended. This would allow for obtaining more robust results and would increase the probability of finding common candidates suitable to be validated in larger cohorts.

Urine is another interesting matrix investigated in patients with breast cancer. In two studies analyzing the same urine samples, the authors first quantified 21 PCs and 12 PEs [50] and then a total of 34 urinary phospholipid molecules belonging to 4 phospholipid classes (12 PSs, 12 PIs, 4 PGs, and 6 PAs) [51]. All phospholipid classes were increased in cancer patients, but a few PIs exhibited some decreases. Although this study suggests that the urine lipid composition of BC patients could be utilized for the possible identification of disease markers, it is remarkable that the general increase of all phospholipid subclasses reported was not observed in studies on plasma and serum.

### 4.2. Gastrointestinal Cancer

Colorectal cancer (CRC) is the second leading cause of cancer-related deaths in the world [33]. It develops from precancerous lesions of the colorectum in 10–15 years. During that period, symptoms are scarce, and early diagnosis is possible via colonoscopy, an invasive procedure. For this reason, there is a need for easily accessible, sensitive, and reliable peripheral biomarkers. In addition, there is evidence that lipid metabolism is strongly related to CRC [52]. Therefore, circulating lipids have attracted considerable attention as potential biomarkers for CRC and over the last 15 years, several studies have investigated plasma and serum lipidome of CRC patients.

In a pioneering investigation, Zhao et al. analyzed lysophospholipid levels in the plasma of 133 patients with CRC and 125 control subjects [53]. Their results showed that a few LPCs, including LPC 18:1 and LPC 18:2, were significantly decreased in CRC patients compared with controls. A few years later, Li et al. reported the analysis of plasma phospholipid levels not only in CRC patients and healthy donors but also in subjects with adenomatous polyps (AP) [54]. It was found that the levels of major LPC species, including 20:3, 18:3, 18:2 and 14:0, were gradually decreased in healthy, AP and CRC samples. Shen et al. investigated a more limited number of samples than the other two studies mentioned above but succeeded in providing a receiver operating characteristic (ROC) curve with an area under the curve (AUC) of more than 0.90 for 8 lipids, including LPC 18:3 and LPC 18:2 [55]. Using serum as biofluid, Rachieriu et al. analyzed the lipid profile of 25 CRC cases classified from stage I to IV and 16 controls, reporting alterations in several subclasses of lipids (PG, PC, PE, PA, FA, CE and Cer), but not in LPC [56]. Potential biomarkers associated with an already developed CRC may be of limited use for its treatment, whereas the detection of precancerous lesions could be more effective in preventing CRC morbidity and mortality. For this reason, Zhu et al. characterized the serum lipidome of 46 subjects carrying a pre-cancerous lesion, advanced colorectal adenoma (CAA), and 50 controls [57]. Although PC 44:5 and PC O-35:6 were increased and showed an outstanding performance for the discrimination of control and CAA, the majority of differentially expressed species belonged to the TAG lipid class, indicating that the abnormal metabolism of TAGs may be involved in CAA formation.

Overall, the studies reported above on plasma lipidomics provide a certain consensus on the decrease in specific LPC species in CRC patients [53,54,55]. Nevertheless, other studies on CRC patient sera reported alternative results [56,57], thus indicating that the methodological approach applied could have an impact on experimental outcomes.

Due to the poor availability of early detection and non-invasive approaches for its diagnosis, there is also an urgent need for gastric cancer (GC) biomarkers. Only a few studies have investigated plasma lipidic alterations in GC so far. In the first one, Saito et al. reported the larger upregulation for PE 36:2 in the GC group, whereas choline-containing phospholipids (PCs, SMs and various LPCs including LPC 22:6, 20:5, 20:0, 18:2 and 22:0) exhibited the most marked down-regulation in the same group [58]. By investigating the progression of pre-cancerous gastric lesions to GC in a prospective targeted lipidomic study, Liu et al. identified 11 plasma lipids decreased during gastric lesion progression to GC, including three PCs, two LPCs, but also lysoPIs (LPI 18:0, LPI 20:4), free FAs (AA, alpha-linolenic acid, stearic acid), and PA 32:1 [59].

Despite the limited number of studies on GC, as in the case of CRC, there is a certain consensus toward a decrease in choline-containing phospholipids in patients compared to controls. Although the physiological/pathological reasons underlining this finding remain elusive, these studies are consistent with others reporting a significant increase in the TAG/phospholipid ratio and a concomitant decrease in total phospholipids in patients with CRC [52], possibly associated with body weight loss and activated inflammatory status. However, these two parameters are not associated with tumors at an early stage; therefore, these biomarkers are possibly of limited use for CRC and GC early detection and stratification.

### 4.3. Prostate Cancer

Prostate cancer (PCa) is the most frequently diagnosed cancer in men worldwide [43]. Although, as in all cancers, early diagnosis is important for the efficacy of treatment, current peripheral biomarkers available, such as PSA, are largely unsatisfactory [34]. Due to the many abnormalities in lipid metabolism in PCa [60], it is not surprising that in recent years, the lipidome of easily accessible body fluids, such as plasma, has been investigated. In 2012, Zhou et al. analyzed 141 PCa patients’ plasma samples and 105 healthy controls, reporting that patients had increased concentrations of all lipid classes except PAs, with the top 15 lipid species discriminating patients and controls containing phosphocholine (LPCs, PCs, SMs) [61]. Chen et al. profiled the plasma lipidome of 30 patients with PCa, 38 with benign prostatic hyperplasia (BPH), and 46 healthy controls, identifying a panel of five lipids able to discriminate between PCa and BPH (all decreased in BPH) and a panel of five lipids distinguishing between the PCa group and healthy controls (PE 32:2 and PS 34:2 increased, while PA 36:3, PE 40:3 and PC 44:2 decreased) [62]. These lipids belong to different subclasses, and LPC species were not included in these panels. In another study, Lin et al. focused their attention on circulating lipids that could be associated with overall survival during docetaxel chemotherapy in castration-resistant prostate cancer (CRPC) [63]. The analysis of 96 patients in the discovery cohort and 63 patients in the validation cohort showed that high levels of circulating sphingolipids were associated with poor prognosis in CRPC, identifying a three-lipid prognostic signature (Cer d18:1/24:1, SM d18:2/16:0 and PC 16:0/16:0) associated with shorter survival. Patel et al. carried out the serum analysis of 57 PCa patients and 76 healthy controls and found a three-lipid signature (PC 38:5, PC 40:3 and PC 42:4) predicting the absence of PCa [64]. These results on PCa show some inconsistencies among studies. Thus, it is evident that additional efforts are needed to standardize sample collection, storage, and analytical methods to obtain more homogenous results. Interestingly, the content of phospholipids in PCa patient urine has not been extensively investigated, with only a single study reporting that four PS and two PI species were differentially expressed in cancer patients [65].

### 4.4. Lung Cancer

The need for reliable and non-invasive biomarkers for lung cancer, allowing early diagnosis of the disease and providing information on the histological phenotype, has stimulated research efforts aimed at investigating the potential use of circulating lipids in plasma and serum. Other matrices, such as saliva and bronchoalveolar lavage, have not been investigated yet, although they could represent an additional and still unexplored source of lipid biomarkers for this pathology. Ravipati et al. analyzed the plasma lipid profile of 17 lung squamous carcinoma and 17 adenocarcinoma patients [66]. They observed that both squamous and adenocarcinomas were characterized by an increase in plasma DAGs and TAGs with respect to healthy controls and a decrease in phospholipids and lysophospholipids. These observations agree with those reported for CRC [52]. Yu et al. succeeded in the identification of four lipid molecules, two up-regulated (LPE 18:1, ether-linked PE PE O-40:4) and two down-regulated (CE 18:2 and SM 22:0) for the early prediction of non-small-cell lung cancer (NSCLC) [67]. Chen et al. analyzed the serum lipidome of early-stage NSCLC, lung benign disease patients and healthy controls, identifying a panel of Pes that were significantly increased in early-stage NSCLC and exhibited good performance in differentiating between early-stage NSCLC and healthy controls [68]. While in tumors of the gastrointestinal tract, there is a certain consensus towards a decrease in LPCs and PCs, in lung cancer, the few available studies draw attention toward PEs, although no common species were identified.

### 4.5. Other Cancers

Lipidomics has also been used to discover circulating biomarkers for the diagnosis and monitoring of other cancer types. Nam et al. compared plasma lipid changes in patients with cervical intraepithelial neoplasia (CIN) of different grades (55 patients with CIN grade 1, 44 with CIN grade 2/3) and 60 patients with cervical cancer (CC) [69]. They found that PCs, PEs, DAGs and free FAs were the lipid classes with more significant differences between patients with high-grade CIN and CC compared to healthy controls and patients with low-grade CIN. In particular, PCs and PEs were decreased in the patients with high-grade CIN and cervical cancer, whereas DAGs and free FAs were significantly increased. The analysis of plasma samples of papillary thyroid carcinomas (47 patients and 33 healthy controls) provided evidence that a few sphingolipids and methyl-PE 18:1/18:1 were up-regulated in cancer patients and could be used as potential diagnostic markers [70]. A recent study investigating plasma lipids for the early detection of malignant gliomas used large discovery (*n* = 107), training (*n* = 750), test (*n* = 225) and validation cohorts (*n* = 920) [71]. The approach led to the identification of a panel of 11 lipids as candidate biomarkers belonging to three lipid classes: three LPCs, which were down-regulated, and seven PCs and TAG 18:1-18:2-18:3, which were up-regulated.

The studies reported above were carried out on different types of tumors and identified diverse molecules, which may reflect an inter-tumoral heterogeneity caused by different lipid metabolism alterations. To unravel whether tumor development is associated with common plasma lipidomic fingerprints, independently from the type of tumor, a few studies compared samples from different cancer types with healthy volunteers. Using a targeted approach to analyze the serum levels of SM 34:1 and five PC species (34:2, 34:1, 36:4, 36:3, and 36:2) in 1449 serum samples, including healthy donors, patients with benign lung, colorectal, gastric and pancreatic diseases, as well as patients with lung, colorectal, gastric and pancreatic cancer, Guo et al. individuated 18 combinations of the six phospholipids having a high diagnostic ability to differentiate between different pathophysiological states [72]. Interestingly, they showed that changes in these phospholipids were significantly correlated with gender. For example, the levels of SM 34:1, PC 34:2, and PC 34:1 in both male and female lung cancer patients were significantly increased, whereas PC 36:3 and PC 36:2 were significantly increased only in male patients with lung cancer. The plasma lipid profile of 5 different cancers (liver, lung, gastric, colorectal, and thyroid) was compared by Lee et al. [73]. Levels of ethanolamine-containing phospholipids, including PEs, LPEs and PE plasmalogens, were significantly low in four cancer types but high in thyroid cancer, thus reinforcing the idea that plasma lipidomics may reveal features common to different types of cancer, as well as cancer-specific features. Wolrab et al. investigated the plasma lipidomic profiles of breast, kidney and PCa patients, finding that the most significant lipid species for cancer separation were CE 16:0, Cer 42:1, LPC 18:2, PC 36:2, PC 36:3, SM 32:1, and SM 41:1, all down-regulated in the plasma of patients as compared to controls, thus representing a potential biomarker panel for breast, kidney and PCa screening [74].

**Table 3:** The table shows the main lipidomic studies of cancer patients’ biofluids and lists the main species changed between cancer and healthy samples or among different stages of the disease. In the study design column, information on age, gender, and food intake prior to sample collection is reported. No information on dietary habits is available for any of these studies. The lipid species reported in the up-regulated and down-regulated column refer to pathological state vs. healthy controls, unless indicated. Relevant individual species are listed for their potential role as biomarkers. The performance of the combination of lipid species or of individual lipids is also reported. Abbreviations: ESI-MS/MS, electrospray ionization tandem MS; LC, liquid chromatography; QqQ, triple quadrupole; NP/RP, normal-phase/ reversed-phase; 2D LC, two-dimensional; HPLC, high-performance liquid chromatography; QTOF, quadrupole time of flight; nLC nanoflow liquid chromatography; UHPLC, ultra-high performance liquid chromatography; N/A, not available.

Biofluid	Study Design	Method	Lipid Species		Performance ^a^	Reference
			Up-Regulated	Down-Regulated		
**Breast cancer (BC)**						
Plasma	-Discovery set: 55 BC patients; 25 age-matched healthy controls; -Training set: 30 BC patients; 20 age-matched healthy controls Gender: Female Food intake: overnight fasting	ESI-MS/MS	PC 34:2	LPC 16:0 PC O-42:5	Sensitivity 98.1%; Specificity 96.0%	Qiu Y. et al., 2013 [44]
Plasma	-Training set: 39 early-stage BC patients (stage 0–II); 51 age-matched benign breast disease patients -Validation set: 45 early-stage BC patients (stage 0–II); 59 age-matched benign breast disease patients Gender: Female Food intake: N/A	LC-ESI-MS/MS (QqQ)	PC 32:1 PC 34:4 PC 38:3 PC 40:5 PC 40:3 PC 44:11 ePC 32:2 ePC 38:3	LPC 18:3 LPC 20:2 LPC 20:1 LPC 20:0 CE 19:1 CE 19:0 CE 20:0	-Validation set: Sensitivity 81.0%; Specificity 94.5%; AUC 0.938	Chen X. et al., 2016 [45]
Plasma	5 BC patients; 6 benign breast disease patients; 9 healthy controls Gender: Female Food intake: N/A	NP/RP 2D LC-MS		PI 32:1 PI 38:4	N/A	Yang L. et al., 2015 [46]
Serum	28 BC patients; 28 benign breast disease patients; 28 age-matched healthy controls Gender: Female Food intake: overnight fasting	UHPLC-MS/MS (QqQ)	PE 30:1 LPE 14:0	PC 36:0 PE 42:2	N/A	More T.H. et al., 2016 [47]
Serum	3 BC patients in stage I invasive ductal carcinoma; 17 in stage II; 11 in stage III; 5 age-matched healthy controls Gender: Female Food intake: N/A	HPLC-ESI-QTOF/ MS	PC (30:3)	DAG 38:9 DAG 34:5	AUC: -0.968 (PC 30:3) -0.910 (DAG 38:9) -0.897 (DAG 34:5)	Socaciu C. et al., 2018 [48]
Urine	5 BC patients, before and after surgery; 5 age-matched healthy controls Gender: Female Food intake: N/A	nLC-ESI-MS-MS	PS 18;1/18:1 PS 18:2/18:0 PC 16:1/16:0 PC 16:0/18:2 PC 18:1/16:1 PC 16:0/16:0 PE 20:0/18:4 PE 16:0/20:4 PE 16:0/18:2	PI 18:0/20:4	N/A	Kim H. et al., 2009 [50] Min H.K. et al., 2010 [51]
**Colorectal cancer (CRC)**						
Plasma	-Training set: 89 CRC patients; 83 healthy controls -Validation set: 44 CRC patients; 42 healthy controls Age: CRC patients 62.3 ± 13.0, controls 46.0 ± 15.8 years Gender: 56% male, 46% female for both groups Food intake: N/A	LC-ESI-MS/MS (QqQ)		LPC 16:0 LPC 18:0 LPC 18:1 LPC 18:2	-Validation set: Sensitivity 82%; Specificity 93%	Zhao Z. et al., 2007 [53]
Plasma	120 CRC patients; 120 adenomatous polyps subjects; 120 age-matched healthy controls Gender: 63% male, 37% female for both groups Food intake: N/A	LC-MS/MS (QqQ)		Saturated LPC sum (14:0, 16:0, 18:0, 20:0, 22:0) LPC 18:1 LPC 18:2	Sensitivity 83%; Specificity 86 %	Li S. et al., 2013 [54]
Plasma	25 CRC patients; 10 healthy controls Age: CRC patients 31–80; controls 18–22 Gender: CRC patients 64% male, 36% female; controls 50% male, 50% female Food intake: overnight fasting	2D LC-QTOF/MS	PG 34:0, SM 42:2 Cer 44:5	LPC 18:3 LPC 18:2 PE O-36:3 PE O-38:3 SM 38:8	AUC: -1.000 (PG 34:0) -0.932 (SM 42:2) -0.916 (Cer 44:5) -0.968 (LPC 18:3) -0.928 (LPC 18:2) -0.936 (PE O-36:3) -0.904 (PE O-38:3) -1.000 (SM 38:8)	Shen S. et al., 2017 [55]
Serum	25 CRC patients; 11 age-matched healthy controls Gender: 63% male, 36% female for both groups Food intake: overnight fasting	UHPLC-ESI- QTOF/MS	Cer 18:0/19:0 PC O-18:0/20:5 PC 18:1/16:0		AUC: -0.941 (Cer 18:0/19:0) -0.888 (PC O-18:0/20:5) -0.885 (PC 18:1/16:0)	Rachieriu C. et al., 2021 [56]
Serum	46 colorectal advanced adenoma patients; 50 age-matched healthy controls: colorectal advanced adenoma patients 58% male, 42% female; controls: 64% male, 36% female Food intake: overnight fasting	UHPLC- MS/MS	PC 44:5 PC O-35:6		AUC: -1.000 (PC 44:5) -1.000 (PC O-35:6)	Zhu Y. et al., 2021 [57]
**Gastric cancer (GC)**						
Plasma	20 GC patients; 16 age-matched controls Gender: GC patients 55% male, 45% female; controls 56% male, 44% female Food intake: N/A	LC-ESI- MS/MS	PE 36:2	LPC 22:6 LPC 20:5 PC 42:9 SM 40:1 PC 36:1 PC 40:7	AUC: -0.744 (PE 36:2) -0.916 (LPC 22:6) -0.844 (LPC 20:5) -0.903 (PC 42:9) -0.884 (SM 40:1) -0.862 (PC 36:1) -0.868 (PC 40:7)	Saito R. et al., 2021 [58]
Plasma	-Discovery set: 31 GC patients; 169 subjects with gastric lesions of different stages; -Validation set: 48 GC patients; 152 subjects with gastric lesions Age: N/A Gender: N/A Food intake: N/A	UHPLC-MS/MS		LPC 18:3 LPC 20:4 PC 38:6 PC 38:5 PC 34:3 LPI 18:0 LPI 20:4 PA 32:1 Free FA 20:4 Free FA 18:3 Free FA 18:0	AUC 0.970	Liu Z.C. et al., 2022 [59]
**Prostate cancer (PCa)**						
Plasma	105 PCa patients; 36 healthy controls Age: N/A Gender: male Food intake: N/A	ESI-MS/MS (QqQ)		LPC 18:1 LPC 20:4 LPC 18:0 LPC 16:0 ePC 38:4 PC 38:4 PC 38:5 PC 40:7 PC 36:1 PC 36:2 SM 18:1 SM 16:1 SM 16:0 SM 18:0 dihydro-SM 16:0	Sensitivity 93.6%; Specificity 90.1%	Zhou X. et al., 2012 [61]
Plasma	30 PCa patients; 38 benign prostatic hyperplasia patients; 46 healthy controls Age: N/A Gender: male Food intake: N/A	LC-ESI-MS/MS (QqQ)	PE 32:2 PS 34:2	PC 44:2 PE 40:3 PA 36:3 LPE 20:0 ^b^ PC 44:5 ^b^ PC 44:2 ^b^ PS 34:2 ^b^ PA 38:3 ^b^	Sensitivity 76.7%; Specificity 80.4% ^b^ Sensitivity 73.3%; ^b^ Specificity 81.6%	Chen X. et al., 2020 [62]
Plasma	-Discovery cohort: 96 castration-resistant prostate cancer (CRPC) patients; -Validation cohort: 63 CRPC patients Age (years, median): discovery cohort 68.9, validation cohort 71.6 Gender: male Food intake: N/A	LC-ESI-MS/MS (QqQ)	Cer d18:1/24:1 SM d18:2/16:0 ^c^ PC 16:0/16:0 ^c^		-Validation set: hazard ratio 4.98	Lin H.M. et al., 2017 [63]
Serum	57 PCa patients; 76 age-matched healthy controls Gender: male Food intake: N/A	ESI-MS/MS (QqQ)	PC 40:3 PC 42:4	PC 38:5	Sensitivity 90.20%; Specificity 86.59%	Patel N. et al., 2014 [64]
Urine	9 PCa patients; 10 healthy controls Age (years): PCa patients 68–83, controls 53–67 Gender: male Food intake: N/A	nLC-ESI-MS/MS	PS 18:0/18:1 PS 16:0/22:6	PS 18:1/18:0 PS 18:0/20:5 PI 18:0/18:1 PI 16:1/20:2	N/A	Min H.K. et al., 2011 [65]
**Lung cancer**						
Plasma	17 squamous cell cancer (SqCC) patients; 17 adenocarcinoma patients; 17 age-matched healthy controls Gender: SqCC patients 76% male, 24% female; adenocarcinoma patients 60% male, 40% female; controls 56% male, 47% female Food intake: N/A	LC-ESI-MS	PS 36:1 SM d18:1/16:0 ^d^		N/A	Ravipati S. et al., 2015 [66]
Plasma	-Discovery set: 60 adenocarcinoma patients; 45 SqCC patients; 80 age-matched healthy controls; -Validation set: 53 adenocarcinoma patients; 41 SqCC patients; 67 age-matched healthy controls Gender: -Training set, 50% male, 50% female for all groups; -Validation set, patients 60% male, 40% female; controls 78% male, 22% female Food intake: N/A	ESI-MS/MS (QqQ)	LPE 18:1 PEO-40:4	CE 18:2 SM 22:0	-Validation set: Sensitivity 78.7%; Specificity 69.4%	Yu Z. et al., 2017 [67]
Serum	66 early-stage non-small-cell lung cancer (NSCLC) patients; 40 benign lung disease (LBD) individuals; 40 age-matched healthy controls Gender of NSCLC patients; 60% male, 40% female; of LBD individuals: 53% male, 47% female; of controls: 60% male, 40% female Food intake: overnight fasting	UHPLC-QTOF-MS/MS	PE 16:0/16:1 PE 16:0/18:3 PE 16:0/18:2 PE 18:0/16:0 PE 17:0/18:2 PE 18:0/17:1 PE 17:0/18:1 PE 20:5/16:0 PE 18:0/18:1 PE 18:1/20:4 PE 18:0/20:3 PC 15:0/18:1 PC 16:1/20:5 PC 18:0/20:1		AUC 0.963	Chen Y. et al., 2018 [68]
**Cervical cancer (CC) and cervical intraepithelial neoplasia (CIN)**						
Plasma	60 cervical cancer patients; 55 CIN patients grade 1; 44 CIN patients grade 2/3; 66 healthy controls Age (years, median): CC patients 50; CIN1 patients 35; CIN2/3 patients 39.5, controls 48 Gender: female Food intake: non-fasting state	UHPLC-QTOF-ESI-MS/MS	DAG 32:0 DAG 32:1 DAG 32:2 DAG 36:4 DAG 36:5 DAG 38:4 DAG 38:5 Free FA 16:1 Free FA 18:1	PE 36:5 PE P-36:2 PE P-36:3 PE P-36:4	N/A	Nam M. et al., 2021 [69]
**Papillary thyroid carcinomas (PTC)**						
Plasma	47 PTC patients; 33 age-matched healthy controls Gender: PTC patients male 23.4%, female 76.6%; controls 18.2% male, 81.8% female Food intake: overnight fasting	UHPLC-QTOF MS/MS	GlcCer d14:1/24:1 SM d16:1/24:1 SM d18:1/15:0 SM d18:1/16:1 methyl-PE 18:1/18:1		Youden index: -0.707 (GlcCer d14:1/24:1) -0.669 (methyl-PE 18:1/18:1) -0.659 (SM d16:1/24:1) -0.651 (SM d18:1/15:0) -0.639 (SM d18:1/16:1)	Jiang N. et al., 2021 [70]
**Glioma**						
Plasma	-Discovery set: 72 brain glioma (BG) patients; 35 age-matched healthy controls; -Training set: 385 BG patients; 365 age-matched healthy controls; -Test set: 115 BG patients; 110 age-matched healthy controls; -Validation set: 351 BG patients; 569 age-matched healthy controls Gender: -Discovery set, BG patients male 60%, female 40%; controls male 47%, female 53% -Training set, BG patients male 60%, female 40%; controls male 47%, female 53% -Test set, BG patients male 63%, female 37%; controls male 49%, female 51% -Validation set, BG patients male 56%, female 44%; controls male 50%, female 50% Food intake: overnight fasting	-Untargeted analysis: LC–MS/MS -Targeted analysis: UHPLC- MS/MS	PC 34:1 PC 34:2 PC 36:4 PC 38:6 PC 36:1 PC 36:2 PC 38:4 TAG 54:6	LPC 16:0 LPC 18:0 LPC 18:2	-Validation set: AUC 0.987	Zhou J. et al., 2022 [71]

^a^ Sensitivity, specificity, and AUC (C.I. 95%) are referred to the combination of all species listed as up-regulated or down-regulated, or to individual species as indicated. ^b^ PCa vs. BHP. ^c^ Associated with shorter survival. ^d^ Adenocarcinoma vs. SqCC.

### 4.6. Final Considerations

Plasma lipidome analysis shows great potential in identifying lipid classes and lipid species, potentially discriminating between cancer patients and healthy controls, as well as between benign and malignant diseases. However, these studies have also shown many limitations. As for other -omics studies, there is a clear reproducibility issue, not only among different types of cancer but also within the same cancer type. Although many studies have focused attention on choline-containing phospholipids, providing evidence of a decrease in LPC and PC species, others have reported increases in PC and SM species. A few investigations have described dysregulated levels of PE species in cancer patients. The sources of this variability may be multiple: diverse criteria in selecting healthy controls, differences in the evaluation of clinical variables or the use of different matrices. For example, plasma and serum are both blood derivatives, but during the preparation of serum, blood clots may release numerous components of platelets, altering the lipid profile. Moreover, the biofluid lipidome may be affected by a number of individual parameters, such as age, gender, body mass index, ethnicity, fasting status and medication, which should be taken into account when analyzing lipidomic data. Therefore, part of the observed result heterogeneity may be due to the lack of information about these parameters. Researchers have often employed various lipid extraction protocols and different technological platforms with different sensitivity, which make study comparison difficult. For instance, the studies listed in Table 3 were carried out using different approaches and often lacked details on the technology used. The number of samples is also a very relevant issue, as some studies are just limited to a few dozen samples, whereas others analyzed hundreds of samples, divided into discovery, training and validation sets. Statistical analysis methods are also often diverse. Finally, another clear limitation of these studies is the inability to identify the cellular/tissue source of the lipids in body fluids. In plasma and serum, most lipids are transported by lipoproteins; nevertheless, circulating EVs also contribute to the lipid profile, but when the whole biofluid is analyzed, EV contributions are often overlooked. Therefore, an intriguing possibility for the development of specific and sensitive lipid biomarkers in liquid biopsies is to characterize the molecular composition of biofluid-derived EVs. Studies addressing this strategy are discussed in the next section.

## 5. Lipidome of EVs in Liquid Biopsies as Potential Cancer Biomarkers

Most studies investigating the molecular cargo of EVs so far have focused on proteins and RNAs. The analysis of EV metabolites, including lipids, has only recently started. Few studies have analyzed the lipid profile of biofluid-derived EVs from cancer patients, despite the promising EV lipidomic data derived from in vitro cell models [75,76,77,78]. Lipid metabolism is altered in cancer cells, and this leads to a different composition not only of cancer vs. normal cells but also of their released EVs. Conversely, knowledge of the lipid cargo carried by EVs, especially those produced by cancer cells, may provide pivotal information regarding the metabolic alterations occurring during oncogenic transformation and might unravel new cancer biomarkers. In this section, we summarize data from studies investigating the lipidome of biofluid-derived EVs, such as plasma/serum and urine, in cancer patients.

### 5.1. Plasma/Serum-Derived EVs

In this paragraph, we report studies showing that several changes in EV lipid profiles were observed in cancer patients. Even if more evidence is needed, the lipidome of blood-derived EVs might potentially become a more reliable and specific source of cancer biomarkers than the whole plasma/serum. In fact, EVs may be enriched from the biofluids, possibly resulting in the enrichment of tumor-associated molecules and, thus, increased biomarker sensitivity and specificity.

A study investigating the lipid profile of EVs isolated from plasma of non-metastatic and metastatic CRC patients and healthy donors indicated that some glycero- and sphingolipid species might be used as diagnostic/prognostic biomarkers of CRC [79]. In particular, PC 34:1, PE 36:2, SM d18:1/16:0, hexosylceramide (HexCer) d18:1/24:0, and HexCer d18:1/24:1, whose levels increased in non-metastatic EVs compared with healthy samples, might be considered potential markers of primary cancer. Moreover, PE 34:2, PE 36:2, alkenyl PE P-16:0/20:4, and Cer d18:1/24:1 were proposed as biomarkers of a metastatic phenotype since the levels of these lipid species were decreased in metastatic patients compared to healthy donors. The lipidome of plasma-derived EVs from patients undergoing colonoscopy has been investigated by Bestard-Escalas and co-workers [80]. Patient cohorts consisted of a healthy group and four pathological groups (patients with hyperplastic polyps, HP; adenomatous polyps, AD; invasive neoplasia, Neo; or hereditary non-polyposis CRC, Her). Decreased levels of molecular species containing only one MUFA (i.e., 34:1, 36:1, and 38:1) and increased levels of species containing PUFA (i.e., 34:2, 36:4, 36:3, 36:2, and 38:4) in the glycerophospholipid classes were observed in EVs from plasma of pathological groups compared with healthy samples. Notably, the ratio between the sum of 34:1-containing species and the sum of 38:4-containing species has the potential to discriminate between healthy donors and patients with a malignant lesion (AD, Neo) or who are prone to have it (Her). These results suggested that MUFAs were substituted by PUFAs in CRC cells, as indicated by studies unveiling an overexpression of enzymes involved in PUFA metabolism in malignant or premalignant lesions [81,82].

Different lipid composition has also been observed in EVs isolated from sera of patients with lung cancer compared to high-risk smokers [83]. An enrichment in Cer 42:1 and a reduced content of PCs containing PUFA were observed in EVs from lung cancer patients [84]. Even if differences in the level of several lipid species were found in serum-derived EVs from lung cancer patients, the high heterogeneity of EV lipid profiles did not allow for clustering samples on the basis of their lipid composition. In fact, the performance of classification models based on EV lipid profile was comparable to the performance of a similar model based on the concentration of metabolites in the whole serum [85].

Sanchez and co-workers identified a specific lipid signature of plasma-derived EVs that allowed the discrimination of patients with cirrhosis and hepatocellular carcinoma (HCC) compared to patients with cirrhosis without HCC [86]. Sphingosines, dilysocardiolipins, lysoPSs and (O-acyl)-1-hydroxy FAs were enriched in EVs from HCC patients compared to non-HCC patients, whereas Sulf and acylGlcSitosterol esters were absent in HCC samples. This study not only identified specific EV lipids as biomarkers of the early stage of HCC but provided information about biological processes occurring in HCC cells that are responsible for the altered lipid asset of released EVs, such as glycerophospholipid metabolism and ferroptosis.

A metabolomic study supported the potential use of serum-derived EVs as a source of biomarkers for pancreatic cancer [87]. EVs were isolated from the serum of pancreatic cancer patients, para-cancerous pancreatic cancer patients and healthy donors. The association analysis between EV lipids and patients’ clinicopathologic characteristics indicated that the EV level of LPC 22:0, PC P-14:0/22:2 and PE 16:0/18:1 correlated with the serum concentration of the tumor markers CA19-9 and CA242 and tumor diameter. Moreover, the EV level of PE 16:0/18:1 was also found to be significantly correlated with the patient’s overall survival [87].

These reports indicate that the altered lipidomes of plasma/serum-derived EVs from cancer patients have the potential to serve as biomarkers for cancer diagnosis and unveil pathological associations between EV lipidomes and tumor progression. However, the limited number of studies investigating the lipid profile of circulating EVs, the diversity of lifestyle and medical conditions of patients, and the variety of methods used for EV isolation and analysis, make the comparison between data from different studies difficult.

### 5.2. Urine-Derived EVs

Urinary EVs (uEVs) are considered a useful and non-invasive clinical diagnostic tool, as many studies have demonstrated that EV molecular content reflects physiological and pathological conditions of the urogenital tract [88]. A few studies have investigated the lipid composition of uEVs isolated from patients affected by renal cell carcinoma (RCC) [89] and PCa [90,91,92].

The first study investigating the lipidome of uEVs demonstrated that RCC patients could be distinguished from healthy subjects based on their EV lipid composition. The levels of several lipid classes were differentially expressed in uEVs from RCC patients; among them, the most relevant change regarded lysophospholipids, whose levels were reduced in RCC samples [89].

Other studies have investigated the lipidome of uEVs in PCa. Skotland and co-workers demonstrated significant changes in the lipid profile of EVs isolated from PCa patients’ urine compared to healthy donors [90]. Among the nine lipid species differently expressed between the two groups, LacCer d18:1/16:0, PS 18:1/18:1 and PS 16:0-18:1 were able to separate PCa and healthy samples with high sensitivity and specificity. These results were quite consistent with a previous study that identified 10 lipid species as potential PCa biomarkers in whole urine; in particular, four PS species (up-regulated 18:0/18:1 and 16:0/22:6; down-regulated 18:1/18:0 and 18:0/20:5) were able to cluster PCa and healthy groups [67]. Lipidomic analysis of two EV fractions isolated from urine using flow field-flow fractionation demonstrated that most phospholipid classes were present at higher levels in the PCa group compared to healthy donors, whereas glycerolipids (DAGs and TAGs) and cholesterol esters were decreased [91]. In uEVs from PCa patients, PS showed the most relevant change, mainly due to PS species containing 16:0, 18:0, and 18:1 [82]. Clos-Garcia and co-workers showed differences in the metabolite content of uEVs isolated from patients with PCa (stages 2 and 3) or BPH [92]. Regarding lipid molecules, reduced levels of several PC species were found in PCa samples compared to BPH. This result, along with studies reporting an increased abundance of PC species in PCa tissue [93], could suggest that fewer PCs are secreted extracellularly via EVs by PCa cells. Another relevant result regarded the different content of free FAs in uEVs from PCa compared with BPH. The abundance of AA, the precursor of eicosanoids and prostaglandins, was decreased in PCa, while other PUFAs with shorter carbon chains (C16:3) were significantly increased [92]. Finally, a univariate analysis comparing PCa stage 2 and stage 3 showed that among the five uEV-associated metabolites whose levels were significantly different between the two groups, four lipids were decreased in stage 3: three Cer species (d18:1/16:0, d18:1/20:0, d18:1/22:0) and PC 30:0. Ceramides are potent regulators of cellular growth and death [94]. Hence, the selective decrease in Cer species in association with a higher disease stage provides an exciting perspective of how this family of metabolites could exert cell and non-cell autonomous functions during the progression of PCa [92].

The results reported in this section indicate that the level of several lipids in uEVs could be utilized as potential biomarkers of tumor detection and progression. In particular, altered levels of PS and ceramide seem to be common features of uEVs from PCa patients. Consistently, the biological relevance of these lipids has to be considered, as PS plays pivotal roles in apoptosis and in the activation of immune system cells [95], and ceramides regulate various aspects of cancer cell biology, including proliferation, survival and cell death [94].

### 5.3. Final Considerations

The studies reported in this section support the idea that the lipidomic profile of biofluid-derived EVs might become a reliable tool for cancer biomarker identification. Nevertheless, the implementation of EV lipidome analysis in a clinical context for cancer diagnosis and prognosis would be possible only if several issues were addressed. One limitation of the use of biofluid-derived EVs as non-invasive tumor biomarkers remains the ability to isolate a pure fraction of cancer-derived EVs from other EV fractions and small particles. Even if the secretion of EVs by tumor cells may be enhanced in some cancer types [96], cancer-derived EVs represent only a small part of vesicles in the biofluids of cancer patients [83]. Moreover, EVs overlap in size and partially in density with lipoproteins such as LDL and VLDL, which are the main transporter of lipids in the blood. Thus, it appears clear that to obtain a reliable EV lipid signature able to differentiate between healthy donors and cancer patients and stratify them according to the stage disease, it is necessary to improve EV isolation methods. The development of diagnostic tools based on the lipidome profiling of circulating EVs deeply relies on this methodological issue.

## 6. Challenges and Possible Solutions for the Use of Lipid Biomarkers in Liquid Biopsies

As presented in this review, the lipidomics of biofluids may provide additional biomarkers for cancer diagnosis. Nevertheless, challenges exist in the translation of such lipidomic data to clinical applications. These are associated with the reproducibility, accuracy, and precision of lipid quantitation. Multiple variables may affect data quality, and quality controls are prerequisites to obtaining reproducible and quantitatively concordant datasets. Therefore, as for other -omics, there is a need to define specific guidelines for study design, sample handling, and data processing, largely accepted by the research community and easily implementable in clinical settings. Recently, McDonald and coworkers suggested the implementation of lipidomic analyses of a dynamic checklist summarizing key experimental details of the studies and using a common language to improve both traceability and reproducibility [97]. In a first attempt to develop guidelines for plasma lipidome analysis, Burla and colleagues pointed out the importance of recording as many pre-analytic parameters as possible since age, gender, body mass index, ethnicity, fasting status and medication may affect the lipidic profile [98]. Appropriate parameter annotation would allow a better data interpretation and decrease the heterogeneity of results. On the other hand, an adequate patient cohort size and multicenter studies would improve statistical power and increase the probability of finding suitable biomarker candidates. Moreover, since different genetic alterations may result in different dysregulated lipid metabolism, inter-tumor lipidome heterogeneity should be expected, as well as intra-tumor differences between tumor stages. Side-by-side genomics and proteomics profiling can help to better understand the significance of lipid level fluctuations and may lead to the development of more refined and defined biomarkers.

In addition to individuals’ variables, many technical aspects may heavily affect lipidic profiling [99]. The importance of controlled and standardized sample preparation and storage should be stressed. Lipids may be subjected to degradation due to the presence of enzymes on biofluids; therefore, sample storage at temperatures below −80 °C is highly recommended [100]. Unsaturated fatty acyl chains may undergo non-enzymatic oxidation in the presence of oxygen; hence, the addition of antioxidants and storage under an inert gas (e.g., nitrogen, argon) may limit the oxidation of the samples. Lipid extraction is the key to ensuring reliable downstream lipidomic analysis, and chloroform/methanol-based protocols such as Bligh and Dyer [101] and Folch [102] have been widely used for a large variety of physiologically relevant lipids. However, they may need to be adapted for complex lipid chemistries and low-abundance and labile metabolites. For instance, these methods present low recoveries for charged and non-polar lipids, such as PA and TAG [103]. Alternative protocols include methyl tert-butyl ether [104] and butanol-methanol [105]. Biofluid lipidomes can be analyzed by a range of MS-based platforms with different sensitivity, resolution, and high-throughput capabilities. The MS instrumentation determines the sensitivity of lipid identification, quantification and annotation. Indeed, lipid extracts may be either directly subjected to MS (shotgun lipidomics) or separated by liquid chromatography (LC) before MS analysis. The chromatographic approach reduces the sample complexity and improves sensitivity. Another important aspect is the ionization method used, as phospholipid head groups have different ionization efficiency. This makes the exact quantification difficult, despite the use of internal standards. In addition, the resolution of the analysis (i.e., the ability to separate molecules with different masses) depends on the mass analyser, i.e., quadrupole, time of flight (TOF) or Orbitrap, interfaced with the ion source. The possibility to annotate a phospholipid species not only by fatty acid sum composition but also by the exact position and identity of the fatty acid acyl chains is provided only by MS/MS. All these aspects must be considered, especially in the description of non-targeted analyses of lipids. Moreover, the lipid amount in the extracts should be taken into account when choosing the MS method for the analysis. For instance, while the more abundant (micromoles to millimoles per liter) lipid classes, such as glycerolipids, glycerophospholipids, sphingolipids and sterols, can be analyzed by more conventional LC-MS/MS, the analysis of very low abundant (nanomoles per liter) lipid mediators (e.g., eicosanoids) requires specialized approaches, such as ultra-high performance liquid chromatography-coupled MS (UHPLC-QTRAP/MS/MS) [106]. Therefore, a truly comprehensive lipidome analysis might require the parallel use of several analytical platforms.

Recent advancements in MS technologies have made possible not only the identification of the lipid molecular species but also the location of the carbon-carbon double bound, sn-position and acyl chain modifications [107]. These methods may provide new means to study lipid metabolism and discover new lipid biomarkers. For example, the relative compositions of the n-9 and n-7 isomers of C18:1 in a variety of PCs and PEs were found to be significantly changed in breast cancer tissue [108]. Nevertheless, many of the techniques are in their infancy, and further development is needed before they become available to the whole research community.

The success of a liquid biopsy approach relies on the ability to differentiate the tumor signal from the surrounding ‘noise’. This can be especially challenging when blood is used as starting material since it naturally contains a large amount of lipids that may mask specific tumor-associated molecules. The analysis of biofluid-derived EVs appears as a promising strategy to increase the signal-to-noise ratio. In fact, even if EVs constitute only approximately 0.005% of the volume of blood plasma [109], tumor-derived EVs may be isolated from biofluids using tumor-specific or tumor-enriched surface markers (e.g., EpCAM), allowing the analysis of biospecimen enriched in tumor-specific cargo. Few studies have demonstrated the feasibility of this approach [110,111,112]; nevertheless, most of the studies so far have focused on the bulk analysis of biofluid-derived EVs. The common methodologies used for EV isolation/enrichment, such as differential centrifugation, size exclusion chromatography and density gradient centrifugation [113], are based on the physical properties of the vesicles. These methods have difficulties separating EV fractions from other small particles present in body biofluids (e.g., virus, lipoproteins, and protein aggregates), thus limiting the sensitivity of EV analysis. Of interest, the total concentration of lipoproteins and protein aggregates in plasma is estimated to be around 10^5^-fold higher than that of EVs [114]. Given the major effort of the scientific community toward the optimization and standardization of these methodological aspects, we envisage the development of diagnostic tools based on circulating EV profiling in the next future. Since EVs are natural carriers for lipids and lipid mediators, the progress of lipidomic approaches in EV-based liquid biopsy is expected.

## 7. Conclusions

In the field of cancer diagnosis, lipidomics has been so far recognized as a discovery tool. However, thanks to advancements in analytic techniques and efforts in the establishment of shared guidelines to ensure data standardization and reproducibility, the field is rapidly expanding into clinical research. In fact, the inventory of lipid molecules found in body fluids offers insights into individual metabolism and physiology and is emerging as a powerful diagnostic and prognostic tool. Therefore, one of the most obvious applications of lipidomics is the targeted profiling of lipids to identify those who are affected by a perturbation of interest and could be used as specific cancer biomarkers. Moreover, as numerous studies have highlighted the intricate relationship between lipid metabolism and cancer development, strategies targeting altered FA and cholesterol synthesis are being explored for cancer therapy. For example, statins (inhibitors of cholesterol synthesis) are currently tested in multiple clinical trials as anti-cancer agents [115], and TVB-2640, an inhibitor of FASN, is currently evaluated in six clinical trials either as a single agent or in combination with others [116]. Targeted lipidomics may provide an indication of patients that may benefit from treatment with lipid synthesis inhibitors and/or used to follow up treatment efficacy.

The findings summarized in this review support the feasibility and potential of biofluid lipidomics for cancer diagnosis. There is consensus on PC and LPC alterations in cancer patients’ liquid biopsies as well as in biofluid-derived EVs. LPC in human plasma can be generated (a) by the activity of phospholipase A2 on PC; (b) by the activity of endothelial lipase, including phospholipase A1, on high-density lipoprotein [117]; or (c) from PC during the formation of cholesteryl esters [118]. However, the molecular mechanisms and the tissues responsible for the observed alteration in PC levels in body fluids have not been established so far.

In conclusion, the studies reported in this review underline the potential use of lipids as tumor biomarkers in liquid biopsies. Optimally, these studies should be focused on the establishment of biomarkers allowing early diagnosis, patient treatment stratification and treatment efficacy follow-up. Moreover, the methodological approach should be fast, cost-effective, and easy to implement in clinical settings. The ongoing efforts aimed at improving both the standardization and reproducibility of lipid quantifications and the sensitivity and specificity of lipid detection foretell the development of lipidomic approaches for the identification of diagnostic and/or prognostic markers in cancer patient’s biofluids in the next future.

## Figures and Tables

**Table 1:** Most common lipid classes in biological membranes. Table adapted from Skotland et al. [11].

Lipid Class/Abbreviation	Sn-1	Sn-2	Headgroup
Phosphatidylcholine/PC	FA ^a^	FA	Choline
Lysophosphatidylcholine /LPC ^b^	FA	H	
Ether-linked PC/ePC (PC O- or PC P-) ^c^	Alkyl or alkenyl	FA	
Phosphatidylserine/PS	FA	FA	Serine
Phosphatidylethanolamine/PE	FA	FA	Ethanolamine
Phosphatidylinositol/PI	FA	FA	Inositol
Phosphatidylglycerol/PG	FA	FA	Glycerol
Phosphatidic acid/PA	FA	FA	H
Ceramide/Cer	LCB ^d^	FA	H
Sphingomyelin/SM	LCB	FA	Phosphocholine
Glycosphingolipids ^e^	LCB	FA	Carbohydrates
Cholesterol/CHOL			
Cholesterol ester/CE	OH group of CHOL esterified with FA		

^a^ FA: Fatty acyl chain. ^b^ Lysophospholipids may be present in all glycerophospholipid classes but, for simplicity, are shown for PC only. ^c^ Ether-linked lipids may be present in all glycerophospholipid classes but, for simplicity, are shown for PC only. Ether-linked lipids with an alkyl group are abbreviated O-, and the ones with an alkenyl group are abbreviated P- and are often called plasmalogens. ^d^ LCB: Long-chain base, often sphingosine (see Figure 1). ^e^ Glycosphingolipids contain many different classes with large variations in their carbohydrate structures.

**Table 2:** Comparison of the advantages and disadvantages of lipid biomarkers versus protein- and nucleic acid-based biomarkers for their potential use in clinical settings. It should be noted that these advantages and disadvantages are dependent to a large extent on the technology platform used. Therefore, the development of more reliable and easy-to-use technologies can change the current view on the pros and cons.

Biomarkers	Advantages	Disadvantages
cfDNA/ cfRNA	- Sample storage and preparation are well-established - Next-generation sequencing (NGS) allows large-scale detection of DNA and RNA molecules and their mutations - RT-PCR is a very well-established method in the clinic - Digital droplet PCR is sensitive, reproducible, relatively inexpensive and suitable for clinical settings	- NSG has a low sensitivity to mutation detection - NGS is costly and complex for clinical settings - PCR has a low multiplexing capacity - Amplification bias limits the power of RT-PCR quantification
Proteins	- MS-based methods allow global protein profiling - Immuno-assay-based technological platforms, such as ELISA, are suitable in clinical settings	- The abundance of proteins in biofluids limits the use of MS for low-expressed proteins - MS is costly and requires specialized instrumentation - Challenges to finding specific and sensitive antibodies for immuno-assays
Lipids	- MS-based methods allow global profiling of cancer-associated lipid alterations - Lipid biomarkers can add new information about diseases and offer new treatment strategies	- MS is costly and requires specialized training and instruments - Lack of rigorous methodological standardization and robust methods for lipid identification
